# Changes in Skeletal Integrity and Marrow Adiposity during High-Fat Diet and after Weight Loss

**DOI:** 10.3389/fendo.2016.00102

**Published:** 2016-07-27

**Authors:** Erica L. Scheller, Basma Khoury, Kayla L. Moller, Natalie K. Y. Wee, Shaima Khandaker, Kenneth M. Kozloff, Simin H. Abrishami, Brian F. Zamarron, Kanakadurga Singer

**Affiliations:** ^1^Division of Bone and Mineral Diseases, Department of Medicine, Washington University, St. Louis, MO, USA; ^2^Department of Molecular and Integrative Physiology, University of Michigan, Ann Arbor, MI, USA; ^3^Department of Orthopaedic Surgery, University of Michigan, Ann Arbor, MI, USA; ^4^Osteoporosis and Bone Biology Division, Garvan Institute of Medical Research, Darlinghurst, Sydney, NSW, Australia; ^5^Division of Pediatric Endocrinology, Department of Pediatrics and Communicable Diseases, University of Michigan Medical School, Ann Arbor, MI, USA; ^6^Graduate Program in Immunology, University of Michigan, Ann Arbor, MI, USA

**Keywords:** obesity, bone, marrow adipose tissue, marrow fat, weight loss, leptin, high-fat diet, fracture

## Abstract

The prevalence of obesity has continued to rise over the past three decades leading to significant increases in obesity-related medical care costs from metabolic and non-metabolic sequelae. It is now clear that expansion of body fat leads to an increase in inflammation with systemic effects on metabolism. In mouse models of diet-induced obesity, there is also an expansion of bone marrow adipocytes. However, the persistence of these changes after weight loss has not been well described. The objective of this study was to investigate the impact of high-fat diet (HFD) and subsequent weight loss on skeletal parameters in C57Bl6/J mice. Male mice were given a normal chow diet (ND) or 60% HFD at 6 weeks of age for 12, 16, or 20 weeks. A third group of mice was put on HFD for 12 weeks and then on ND for 8 weeks to mimic weight loss. After these dietary challenges, the tibia and femur were removed and analyzed by micro computed-tomography for bone morphology. Decalcification followed by osmium staining was used to assess bone marrow adiposity, and mechanical testing was performed to assess bone strength. After 12, 16, or 20 weeks of HFD, mice had significant weight gain relative to controls. Body mass returned to normal after weight loss. Marrow adipose tissue (MAT) volume in the tibia increased after 16 weeks of HFD and persisted in the 20-week HFD group. Weight loss prevented HFD-induced MAT expansion. Trabecular bone volume fraction, mineral content, and number were decreased after 12, 16, or 20 weeks of HFD, relative to ND controls, with only partial recovery after weight loss. Mechanical testing demonstrated decreased fracture resistance after 20 weeks of HFD. Loss of mechanical integrity did not recover after weight loss. Our study demonstrates that HFD causes long-term, persistent changes in bone quality, despite prevention of marrow adipose tissue accumulation, as demonstrated through changes in bone morphology and mechanical strength in a mouse model of diet-induced obesity and weight loss.

## Introduction

Over the past two decades, the prevalence of obesity has increased in Western countries (1, 2). In the United States, currently ~68.6% of adults and approximately one-third (~31.8%) of children are overweight or obese (3). Obesity is associated with comorbidities including cardiovascular and metabolic disease, autoimmune disorders and some cancers (1, 4–6). Recent work has suggested that obesity is also detrimental to bone health (7–11), with skeletal changes that can persist even after weight loss (10, 12).

Previously, it was assumed that obesity had a purely positive effect on bone mass (13–15); increased body weight provides mechanical stimulation, resulting in skeletal loading and bone accrual. However, juxtaposed to this, there is a newly recognized metabolic component, as the adipose tissue itself can exert a negative influence on bone (14). Indeed, increases in body mass index (BMI) have been associated with decreased bone mineral density (BMD) and increased fracture risk in obese adolescents and adults (9, 16), and in obese children (17). The effect of obesity on fracture risk is site specific. The presence of soft-tissue padding from fat may contribute to decreased fracture risk in some areas (e.g., hip) while unprotected sites, such as the extremities (e.g., humerus and ankle), have increased risk (18–20).

The cross-sectional nature of previous clinical studies can only identify associations between obesity and bone, thus, rodent models are widely utilized to explore the mechanisms underlying the relationship between obesity and the skeleton. It is well established that high-fat feeding of mice leads to a reduction in cancellous bone mass (7, 12, 21, 22). This may be mediated by leptin-induced sympathetic tone, which has been implicated as strong mediator of cancellous bone loss (23–25). By comparison, the cortical phenotype in response to high-fat diet (HFD) in rodents remains unclear, with some studies indicating an increase (11), no change (12, 21, 22, 26) or a reduction in cortical bone mass (10, 27). Located within the skeleton are the bone marrow adipocytes; recent studies suggest that marrow adipose tissue (MAT) expansion occurs during high-fat feeding (28, 29). Whether MAT expansion and bone loss are somehow linked during obesity is still unclear; some studies suggest that these lineages are correlated (29–31) while Doucette et al. recently reported MAT expansion during diet-induced obesity that occurred independently of a bone phenotype (28).

In addition to the effects of obesity on bone, weight loss interventions have also been shown to have detrimental effects on bone metabolism, as reviewed by Brzozowska et al. (32). There are a range of interventions including calorie-restricted diets, exercise regimens, medications, and bariatric surgery (32, 33). Each of these interventions aim to reduce body fat and improve metabolic disease; the full extent to which these processes may alter MAT and bone mass in the context of obesity are largely unknown. Surgical interventions of bariatric surgery (Roux-en Y gastric bypass, laparoscopic adjustable gastric banding, and sleeve gastrectomy) have all been associated with a decline in bone mass despite improvements in metabolic health (32). In contrast to surgical weight loss, exercise has been shown to be quite beneficial on bone density due to increased muscle loading (34–36). The most common initial intervention clinically is calorie restriction or “dieting.” Few studies have looked at weight loss in rodent models through interventions of “switching” diet. One study performed showed that switching back to a chow diet following high-fat feeding could rescue bone loss (12); however, the response of MAT and the interaction of MAT with bone loss in these models was not examined.

The objective of this study was to investigate the interaction between MAT and bone in the context of high-fat feeding and to examine the response of these tissues to dietary weight loss. We demonstrate that high-fat feeding leads to excess peripheral adiposity, MAT expansion, a reduction in bone mass and impaired bone strength. Weight loss led to a significant reduction in whole body adiposity and blocked MAT expansion; however, it failed to completely rescue defects in skeletal morphology and biomechanics. This work begins to address the potential of adipose tissue within the skeleton to have an impact on bone – working, unlike peripheral fat, from the inside out.

## Materials and Methods

### Animals

Male C57Bl6/J mice (Jackson Laboratories) were given a normal chow diet (ND) (13.5% calories from fat; LabDiet 5LOD) or 60% high fat diet (HFD) (Research Diets D12492) at 6 weeks of age for a duration of 12, 16, or 20 weeks. A third group of mice was put on HFD for 12 weeks and then on ND for 8 weeks [weight loss (WL) group]. Animals were housed in a specific pathogen-free facility with a 12-h light/12-h dark cycle at ~22°C and given free access to food and water. All animal use was in compliance with the Institute of Laboratory Animal Research Guide for the Care and Use of Laboratory Animals and approved by the University Committee on Use and Care of Animals at the University of Michigan. The tibia was selected for our longitudinal analyses since it can be used to simultaneously monitor changes in rMAT (proximal tibia) and cMAT (distal tibia) within one sample (37). To compare the changes in bone within the tibia to those in the femur, as reported previously (12), we also analyzed the femurs in the 20-week groups.

### Micro Computed-Tomography

Tibiae were fixed in formalin for 48-h and then placed in phosphate buffered saline (PBS). Specimens were embedded in 1% agarose and placed in a 19-mm diameter tube, and the length of the bone was scanned using a Micro Computed-Tomography (microCT) system (μCT100 Scanco Medical, Bassersdorf, Switzerland). Scan settings were: voxel size 12 μm, medium resolution, 70 kVp, 114 μA, 0.5 mm AL filter, and integration time 500 ms. Density measurements were calibrated to the manufacturer’s hydroxyapatite phantom. Analysis was performed using the manufacturer’s evaluation software.

Femurs were removed and frozen after wrapping in PBS-soaked gauze and then analyzed by microCT. Femora were scanned in water using cone beam computed tomography (explore Locus SP, GE Healthcare Pre-Clinical Imaging, London, ON, Canada). Scan parameters included a 0.5° increment angle, four frames averaged, an 80 kVp and 80 μA X-ray source with a 0.508 mm AI filter to reduce beam hardening artifacts, and a beam flattener around the specimen holder. All images were reconstructed and calibrated at an 18 μm isotropic voxel size to manufacturer-supplied phantom of air, water, and hydroxyapatite (38).

### Biomechanical Assessment

Following microCT scanning, femurs were loaded to failure in four-point bending using a servohydraulic testing machine (MTS 858 MiniBionix, Eden Prairie, MN, USA). All specimens were kept hydrated in lactated ringers solution-soaked gauze until mechanical testing. In the same mid-diaphyseal region analyzed by μCT, the femur was loaded in four-point bending with the posterior surface oriented under tension. The distance between the wide, upper supports was 6.26 mm, and the span between the narrow, lower supports was 2.085 mm. The vertical displacement rate of the four-point bending apparatus in the anterior–posterior direction was 0.5 mm/s. Force was recorded by a 50 lb load cell (Sensotec) and vertical displacement by an external linear variable differential transducer (LVDT, Lucas Schavitts, Hampton, VA, USA), both at 2000 Hz. A custom MATLAB script was used to analyze the raw force-displacement data and calculate all four-point bending parameters. Combining anterior–posterior bending moment of inertia data from μCT with mechanical stiffness from four point bending, the estimated elastic modulus was calculated using standard beam theory as previously described (38). The modulus of elasticity was derived based on previous methods with “L” set at 3.57 and “a” at 0.99 (39).

### Quantification of Trabecular and Cortical Parameters with microCT

*Tibia*. Regions of interest (ROI) was located for both cortical and trabecular parameters. Analyses were performed with MicroCT software provided by Scanco Medical (Bassersdorf, Switzerland). A mid-diaphyseal cortical ROI was defined as ending at 70% of the distance between the growth plate and the tibia/fibula junction. A ROI spanning 360 μm (30-slices) proximal to this region was analyzed with standard plugins using a threshold of 280. The trabecular ROI was defined as starting 60 μm (5-slices) distal to the growth plate and ending after 600 μm total (50-slices). Trabecular analyses were performed with standard Scanco plugins with a threshold of 180.

*Femur*. ROI was located for both cortical and trabecular parameters. A diaphyseal cortical ROI spanning 18% of total femur length was located midway between the distal growth plate and third trochanter. Cortical bone was isolated with a fixed threshold of 2000 Hounsfield Units for all experimental groups. Parameters including cortical thickness, endosteal and periosteal perimeter, cross sectional area, marrow area, total area, anterior–posterior bending moment of inertia, and tissue mineral density (TMD) were quantified with commercially available software (MicroView v2.2 Advanced Bone Analysis Application, GE Healthcare Pre-Clinical Imaging, London, ON, Canada). A trabecular ROI 10% of total femur length was located immediately proximal to the distal femoral growth plate and defined along the inner cortical surface with a splining algorithm. Trabecular metaphyseal bone was isolated with a fixed threshold of 1200 Hounsfield Units.

### Quantification of Marrow Adipose Tissue

Marrow adipose tissue volume within the tibia was assessed as described previously (37, 40). After the initial microCT scan, bones were decalcified in 14% EDTA solution, pH 7.4 for 14 days at 4°C. Decalcified bones were stained with 1% osmium tetroxide solution in Sorensen’s phosphate buffer pH 7.4 at room temperature for 48 h. Osmium-stained bones were re-scanned using the Scanco microCT settings described above. For analysis of MAT within the tibia, four regions were defined as follows: (1) the proximal epiphysis between the proximal end of the tibia and the growth plate, (2) the proximal metaphysis, beginning 60 μm (5-slices) distal to the growth plate and ending after 600 μm total (50-slices), (3) the growth plate to the tibia/fibula junction (GP to T/F J), and the distal tibia between the tibia/fibula junction and the distal end of the bone. MAT volume analyses were performed with standard Scanco plugins with a threshold of 500.

### Statistics

**Figure 1:**
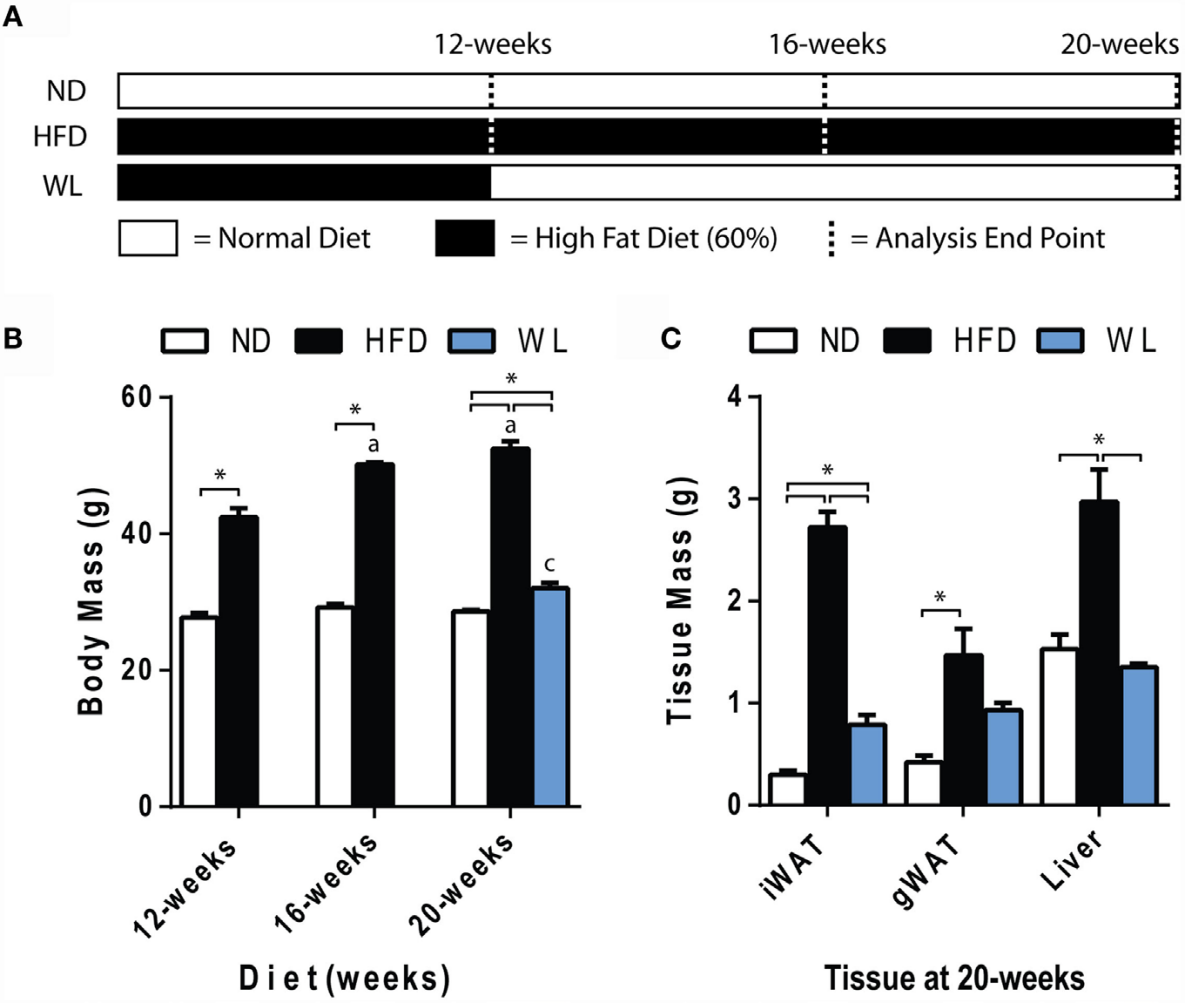
**Body and tissue mass**. **(A)** Experiment outline. Starting at 6-weeks of age, mice were fed the indicated diets for up to 20 weeks prior to analysis. Seven groups of mice were analyzed as indicated by the dashed lines. ND: normal chow diet; HFD: high fat diet; WL: weight loss. **(B)** Body mass. *N* = 7-8 per group. **(C)** Tissue mass at 20 weeks. *N* = 4-6 per group. All graphs are mean ± SEM. “a” – significant vs. 12-week on same diet. “b” – significant vs. 16-week on same diet. “c” – significant vs. 12-week HFD. *p < 0.050 for the indicated comparison.

**Figure 2:**
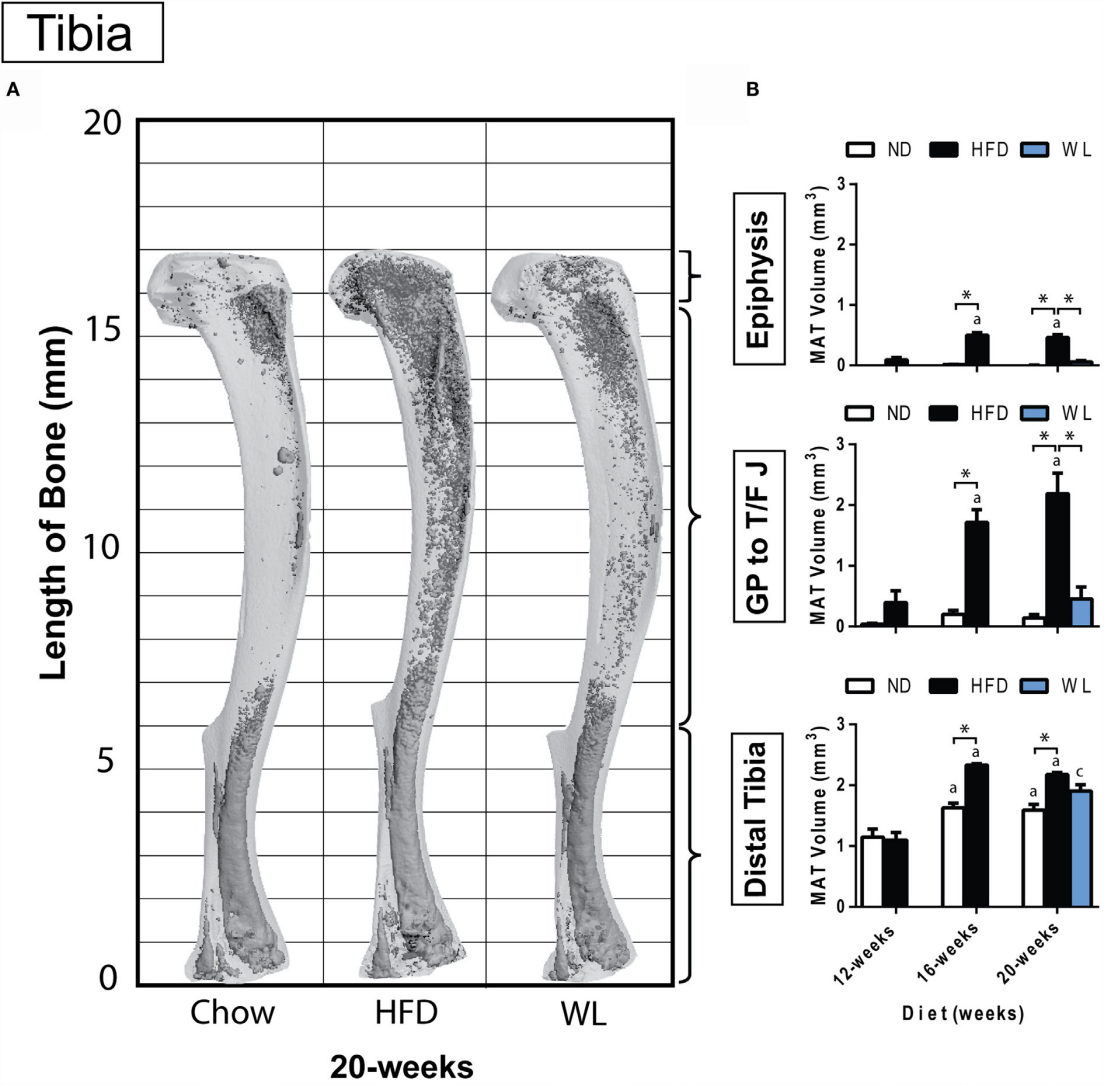
**Region-specific changes in MAT volume with HFD and WL**. **(A)** 3D reconstruction of osmium tetroxide stained MAT (dark gray) overlaid on the tibia bone (light gray). **(B)** Region-specific quantification of MAT in the proximal tibial epiphysis, between the growth plate to tibia/fibula junction (GP to T/F J), and the distal tibia. All graphs are mean ± SEM. *N* = 3-6 per group for the proximal epiphysis; low *N* is due to accidental removal/fracture of the proximal epiphysis during processing. *N* = 6–8 for all other groups. ND, normal chow diet; HFD, high-fat diet; WL, weight loss. “a” – significant vs. 12-week on same diet. “b” – significant vs. 16-week on same diet. “c” – significant vs. 12-week HFD. *p < 0.050 for the indicated comparison.

**Figure 3:**
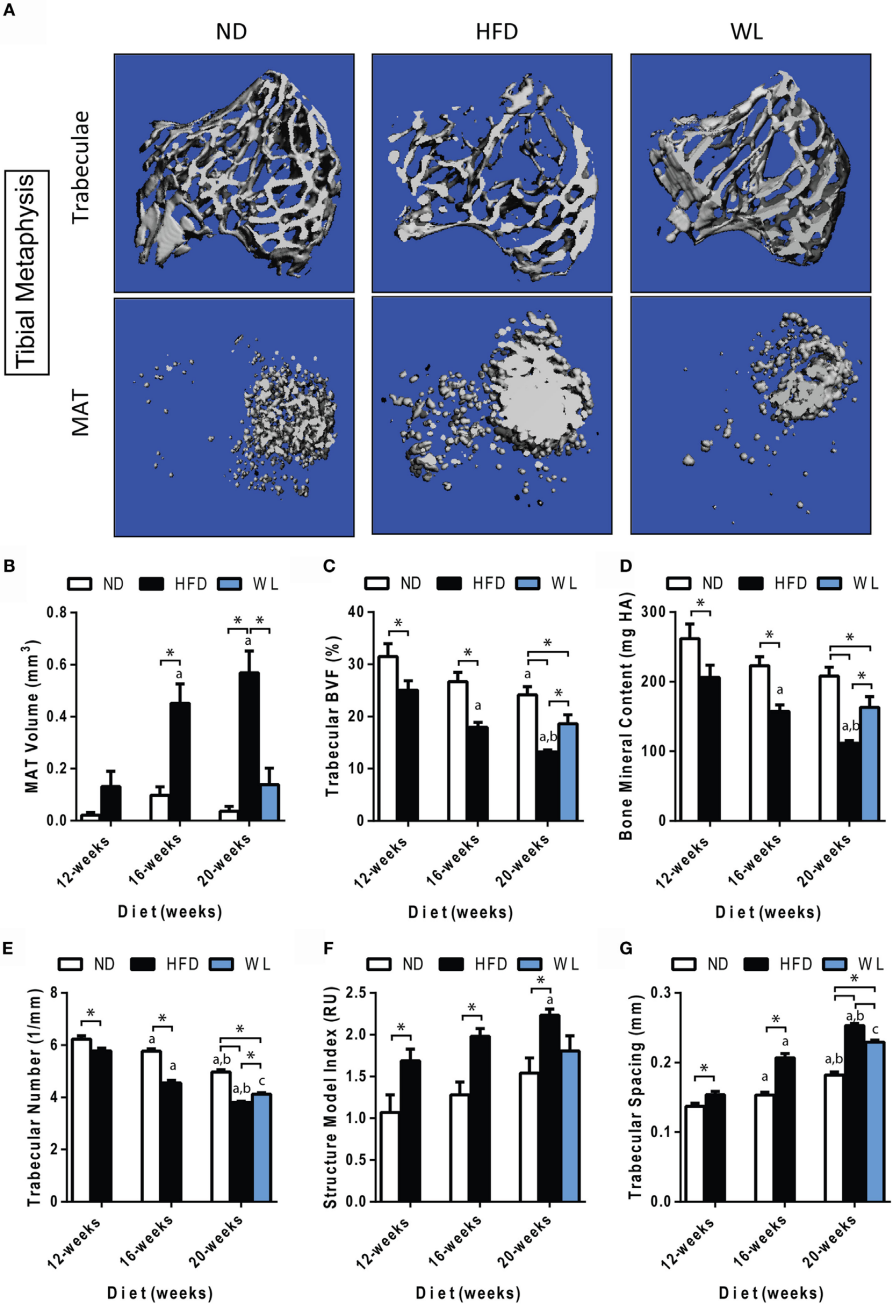
**Changes in MAT and trabecular architecture at the proximal tibial metaphysis over time**. **(A)** Representative images of trabecular architecture and MAT at the 20-week time point. Images of trabeculae and MAT are from the same bone for a given diet. **(B–G)** Quantification of MAT and trabecular parameters in the proximal metaphysis of the tibia. All graphs are mean ± SEM. *N* = 7–8 per group. ND: normal chow diet; HFD: high-fat diet; WL: weight loss. “a” – significant vs. 12-week on same diet. “b” – significant vs. 16-week on same diet. “c” – significant vs. 12-week HFD. *p < 0.050 for the indicated comparison.

**Figure 4:**
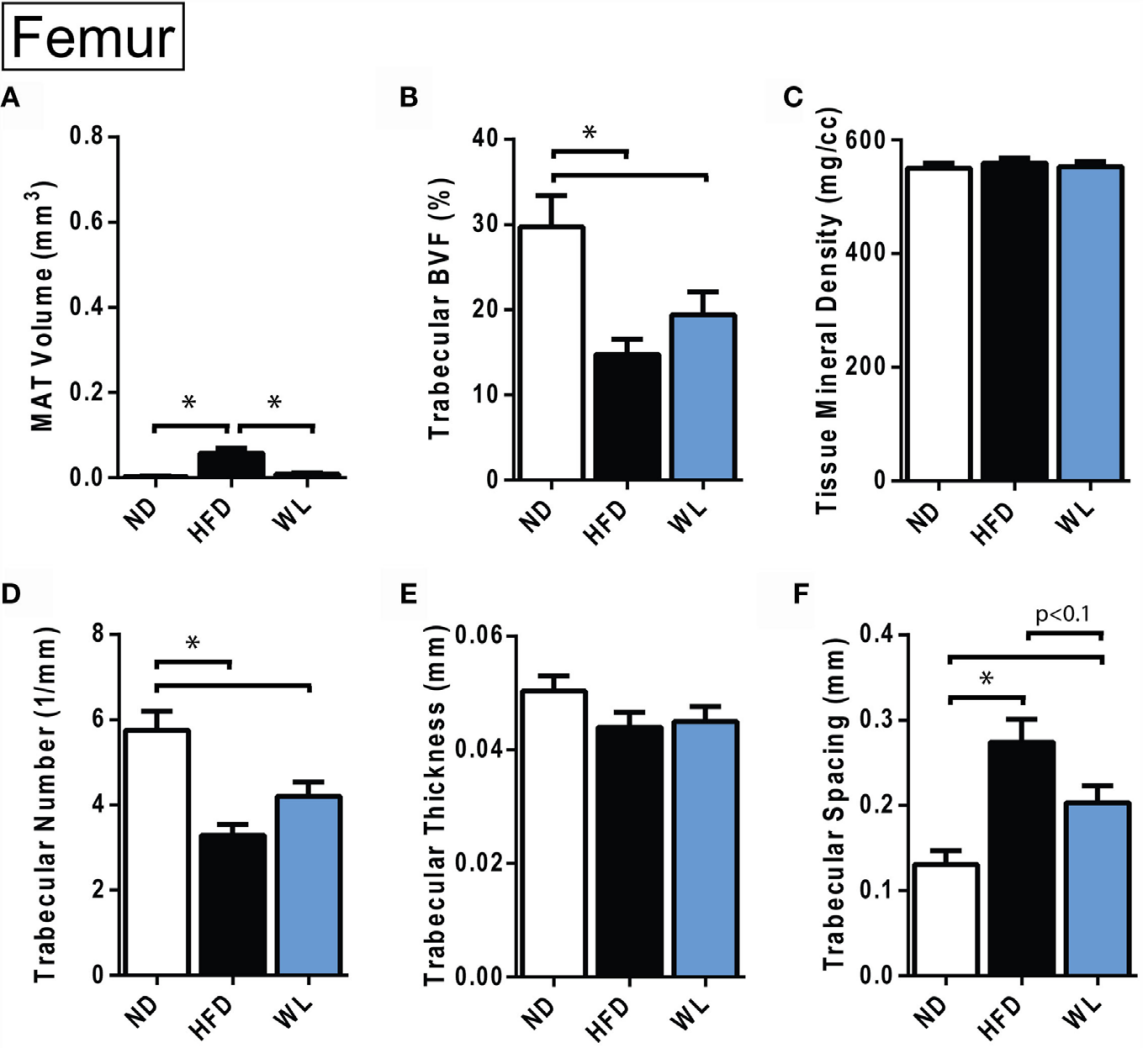
**Changes in MAT and trabecular architecture at the distal femoral metaphysis at 20-weeks**. **(A–F)** Quantification of MAT and trabecular parameters in the distal metaphysis of the femur. All graphs are mean ± SEM. *N* = 8 per group. ND, normal chow diet; HFD, high-fat diet; WL, weight loss. *p < 0.050 for the indicated comparison.

**Figure 5:**
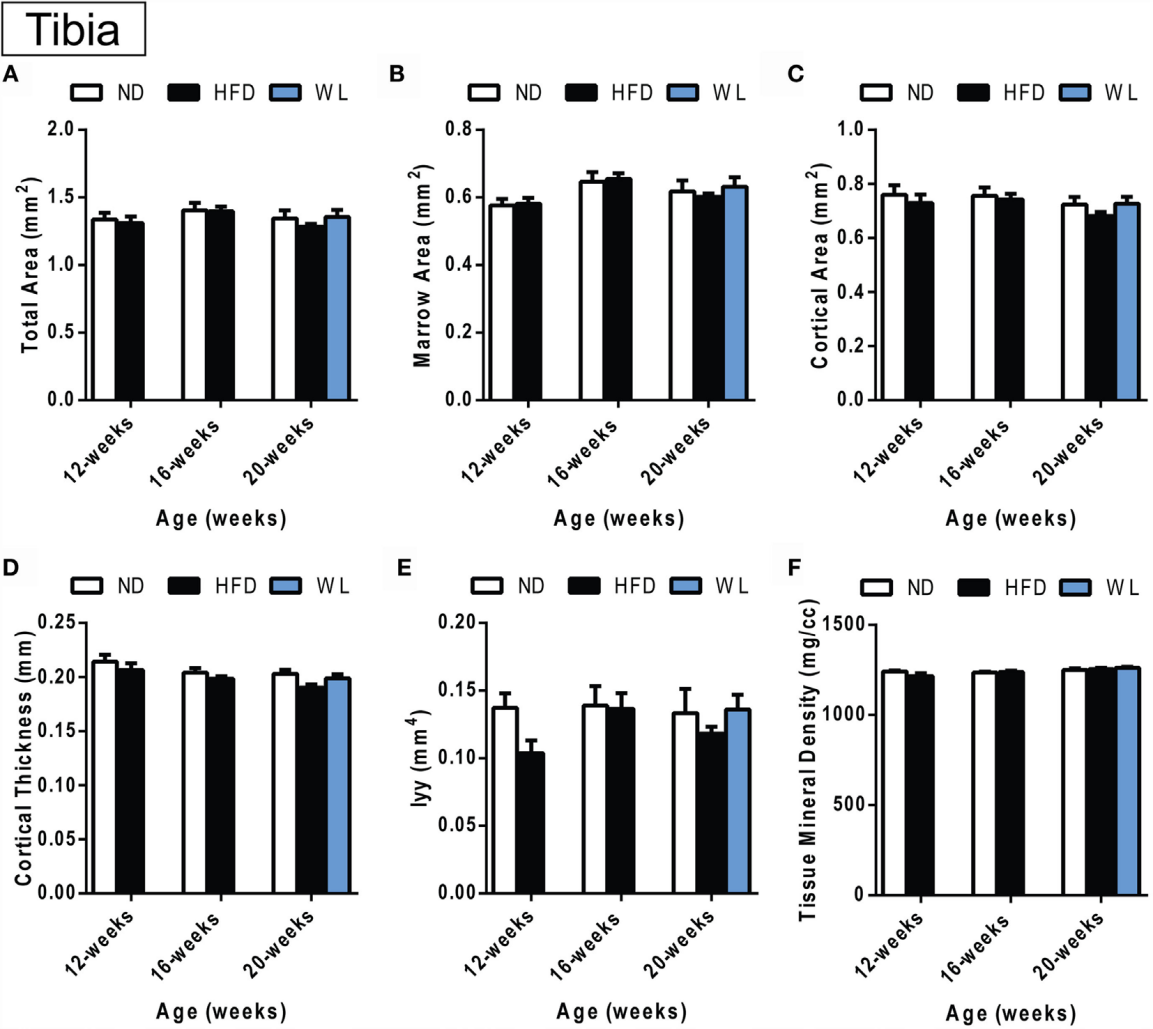
**Changes in cortical morphology at the mid-tibial diaphysis**. **(A–F)** Quantification of cortical parameters in the mid-diaphysis of the tibia. *N* = 7–8 per group. ND: normal chow diet; HFD: high-fat diet; WL: weight loss. All graphs are mean ± SEM. “a” – significant vs. 12-week on same diet. “b” – significant vs. 16-week on same diet. “c” – significant vs. 12-week HFD. *p < 0.050 for the indicated comparison.

**Figure 6:**
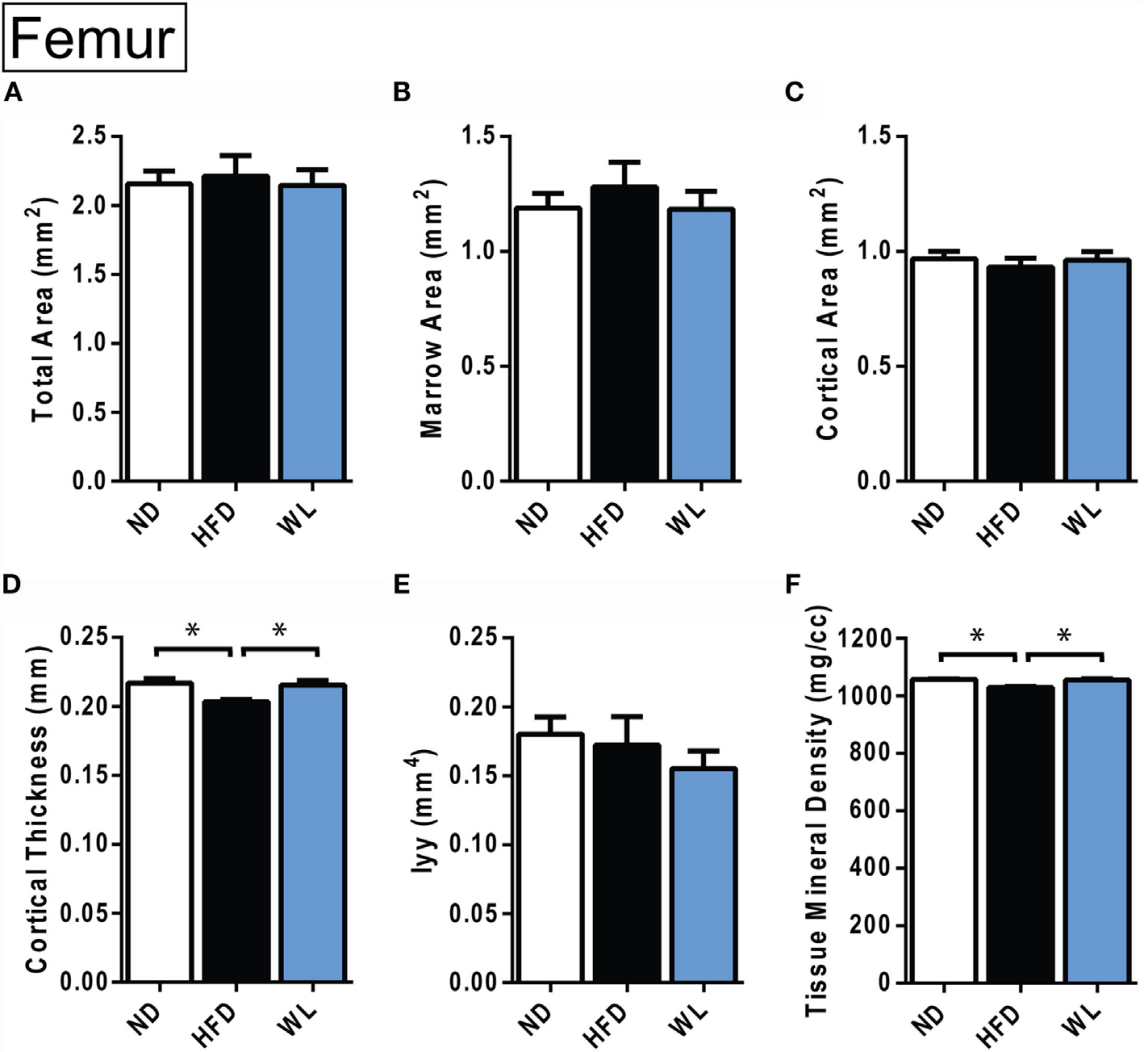
**Changes in cortical morphology at the mid-femoral diaphysis**. **(A–F)** Quantification of cortical parameters in the mid-diaphysis of the femur. *N* = 8 per group. ND, normal chow diet; HFD, high-fat diet; WL, weight loss. All graphs are mean ± SEM. *p < 0.050 for the indicated comparison.

**Figure 7:**
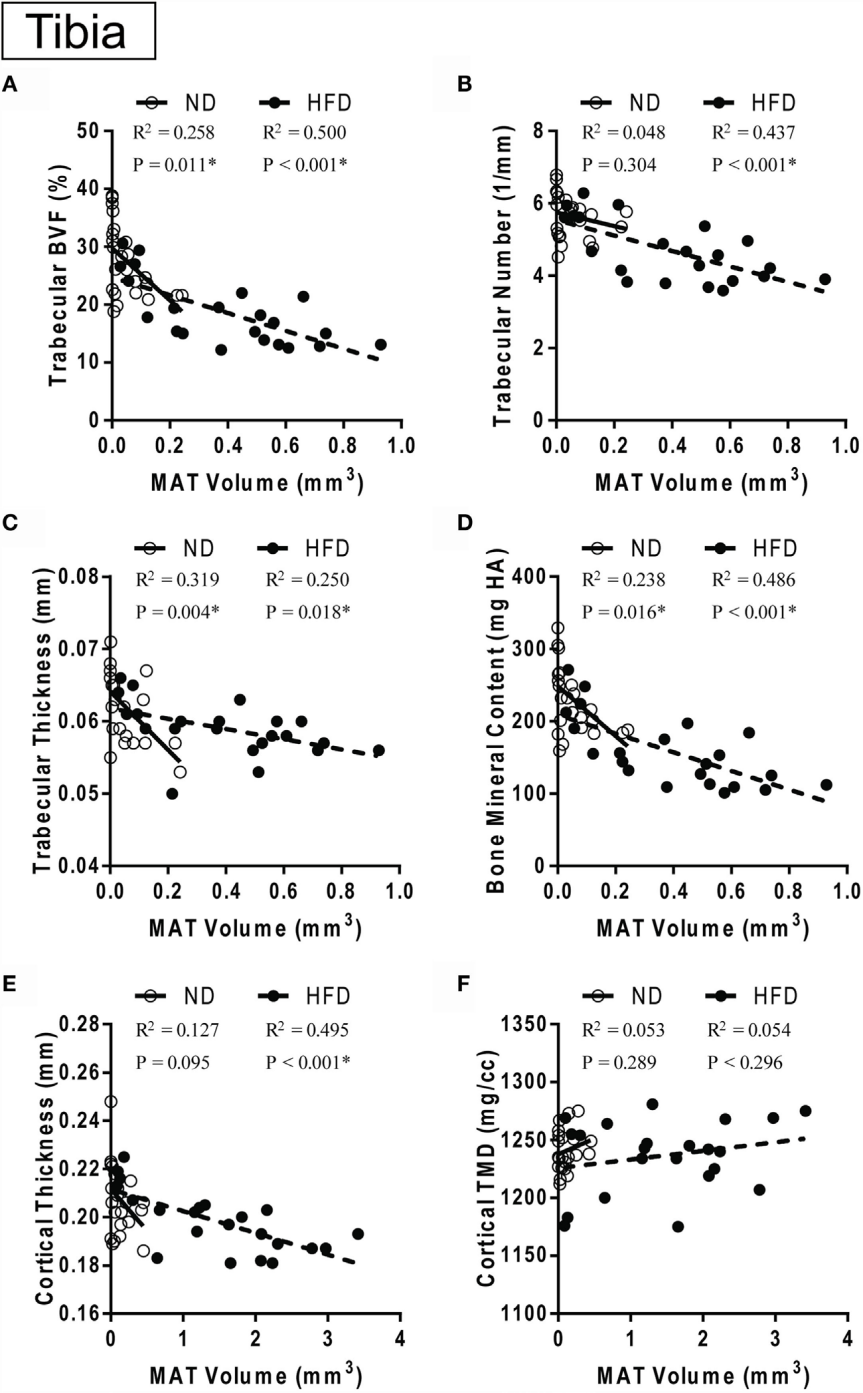
**MAT vs. bone over time with control or high-fat diet**. **(A–D)** Correlation between marrow adipose tissue volume in the proximal metaphysis and trabecular parameters. **(E,F)** Correlation between marrow adipose tissue volume between the growth plate and tibia/fibula junction and cortical parameters in the same bone. Data grouped by diet type. ND: normal diet control for 12, 16and 20 weeks. HFD: high-fat diet for 12, 16, and 20 weeks. Linear regression. *p < 0.050 for the indicated diet.

**Figure 8:**
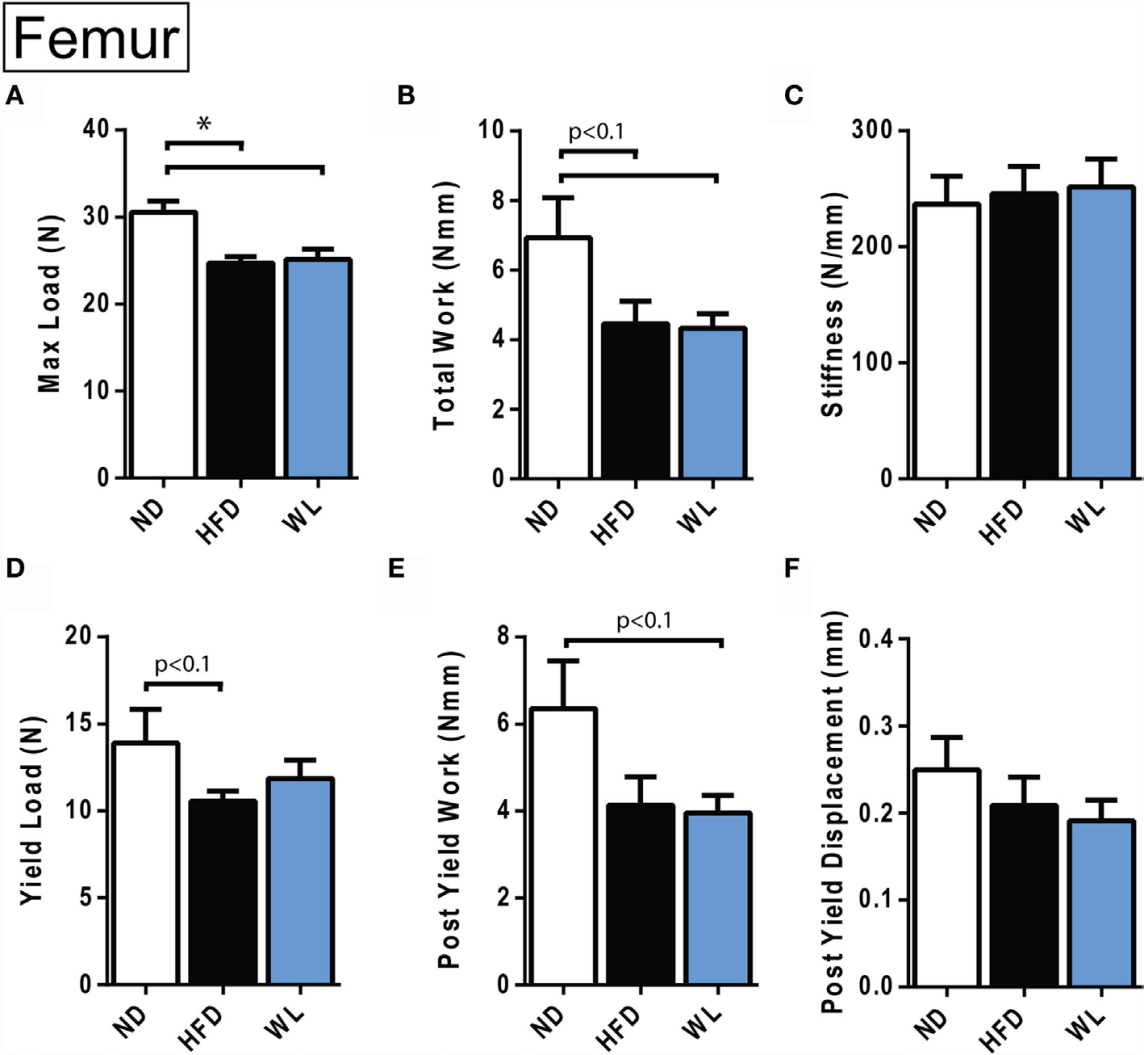
**Femur biomechanical testing at 20 weeks**. **(A–F)** Quantification of biomechanical parameters, as assessed by four-point bending. *N* = 12–14 per group. ND, normal chow diet; HFD, high-fat diet; WL, weight loss. All graphs are mean ± SEM. *p < 0.050 for the indicated comparison.

Statistical comparisons were performed in GraphPad Prism (GraphPad Software, Inc., La Jolla, CA, USA). The following planned comparisons were performed on the graphs in Figures 1, 2, 3 and 5: 12-week ND vs. HFD (two-tailed *t*-test); 16-week ND vs. HFD (two-tailed *t*-test); 20-week ND vs. HFD vs. WL (1-way ANOVA); 12-, 16-, 20-week ND (1-way ANOVA); 12-, 16-, 20-week HFD (1-way ANOVA); 12-week HFD vs. 20-week WL (two-tailed *t*-test). These results were corrected for multiple comparisons using the Benjamini-Hochberg procedure as described previously (41). For comparisons in Figures 4, 6 and 7, a one-way ANOVA with Tukey’s correction was applied. In Figure 8, linear regression was applied to test the significance of the correlations. Raw data for the skeletal morphology, marrow fat quantification, and biomechanical testing is available in Data Sets 1–3 in Supplementary Material.

## Results

### Increases in Body Mass with High-Fat Diet Are Rescued by Weight Loss

Mice were fed normal chow diet (ND) or 60% high-fat diet (HFD), starting at 6 weeks of age, for 12, 16, or 20 weeks. A separate group of mice received 12 weeks of HFD, followed by 8 weeks of ND to mimic weight loss (Figure 1A). Comparison of the 12-week HFD group to the WL group was used to determine if weight loss reversed changes that were already present at 12 weeks, or, rather, prevented further deterioration induced by continued HFD.

Increases in body mass relative to ND control were apparent after 12-weeks of HFD (Figure 1B). Increases in body mass relative to ND control persisted at the 16- and 20-week time points (Figure 1B). Over time, body mass continued to increase from 12 to 16 weeks of HFD but stabilized between 16 and 20 weeks (Figure 1B). Body mass returned to normal after weight loss (Figure 1B). Weight gain was due, at least in part, to increases in liver, inguinal and gonadal white adipose tissue (WAT) mass after 20-weeks of HFD (Figure 1C). With weight loss, liver mass returned to normal; however, WAT mass was only partially rescued (Figure 1C).

### Marrow Adipose Tissue Expansion after High-Fat Diet Feeding Is Inhibited with Weight Loss

Within the tibia, MAT expansion became significant, relative to ND control, after 16 weeks of HFD (Figures 2A,B). With HFD, changes in the proximal tibial epiphysis mimicked what was observed between the growth plate and tibia/fibula junction, with a 5.5- and 4.3-fold increase at 16 weeks, relative to 12 weeks, respectively (Figure 2B). The distal tibia was similar, though there was only a 2.1-fold increase between 12 and 16 weeks of HFD, likely owing to the higher baseline MAT in this region (Figure 2B). No additional MAT accrual in any region of the tibia was observed between 16 and 20 weeks of HFD (Figure 2B).

In the weight loss group, the MAT in the regions of the proximal epiphysis and GP to T/F J was indistinguishable from that of the 20-week ND group (Figure 2B). However, it was also similar in magnitude to the 12-week HFD group, suggesting that switching to ND was sufficient to block HFD-induced MAT expansion – rather than reversing MAT accrual that had already occurred.

In the distal tibia, age-associated increases in MAT were noted from 12- to 16-weeks of age in the ND group. Prior to correction for multiple comparisons, MAT within the distal tibia in the WL group was higher than chow (*p* = 0.019) but less than HFD (*p* = 0.050). This suggests that weight loss blunted, but did not entirely prevent, HFD-induced MAT expansion in the distal tibia at 20-weeks (Figure 2B). Raw data for these comparisons is available as Data Set 1 in Supplementary Material.

### In the Tibia, Trabecular Bone Quality Decreases with High-Fat Diet and Partially Improves with Weight Loss

Consistent with previous reports (42, 43), we observed an age-related decrease in trabecular bone volume fraction (BVF) and trabecular number, with a corresponding increase in spacing, in the proximal tibial metaphysis of ND control mice (Figures 3A,C,E,G).

Relative to ND controls, mice fed a HFD had a significant decrease in trabecular BVF, bone mineral content (BMC), and number after 12-weeks of diet (Figures 3C,D,E). Structure model index and trabecular spacing were reciprocally increased (Figures 3F,G). Trabecular thickness remained unchanged (Data Set 1 in Supplementary Material). Loss of trabecular BVF and BMC with HFD, relative to control ND, persisted at the 16- and 20-week time points (Figures 3C–G). Weight loss partially rescued decreases in trabecular BVF, BMC, and number (Figures 3C–E) and increases in spacing (Figure 3G).

Unlike loss of trabecular bone at 12-weeks, MAT volume was not significantly increased relative to ND control until 16- and 20-weeks of HFD (Figure 3B). Over time, MAT increased by 3.5-fold from 12- to 16-weeks of age in the HFD group (Figure 3B). No further increases were present from 16- to 20-weeks of age. Weight loss completely prevented HFD-induced MAT accumulation from 12- to 20-weeks (Figure 3B).

### In the Femur, Trabecular Bone Quality Decreases with High-Fat Diet and after Weight Loss

Relative to ND control, HFD caused MAT expansion within the femur (Figure 4A). MAT expansion was absent in the WL group (Figure 4A). The absolute amount of MAT in the distal femoral metaphysis was ~90% less than the proximal tibia (*p* < 0.0001, *t*-test) (Figures 3B and 4A). The magnitude of the loss of BVF with HFD at 20-weeks was comparable between femur and tibia (50 vs. 45%) (Figure 4B; Data Sets 1 and 2 in Supplementary Material). There was also a significant, HFD-induced decrease in trabecular number and increase in trabecular spacing (Figures 4D,F). Trabecular thickness and tissue mineral density remained unchanged (Figures 4C,E). With weight loss, there were no statistically significant differences in trabecular morphology, relative to HFD, in the femur (Figures 4B–F). There was a non-significant trend toward a decrease in trabecular spacing in the WL group relative to HFD (Figure 4F).

### Changes in Cortical Bone after High-Fat Diet and Weight Loss

Within the tibia, there were no statistically significant changes in mid-diaphyseal cortical morphology after 12, 16, or 20 weeks of HFD or after weight loss (Figures 5A–F). However, slight differences may have been missed after statistical correction for multiple comparisons. For example, with standard one-way ANOVA at the 20-week time point only, there was a slight decrease in cortical thickness in the 20-week HFD group relative to ND control (*p* = 0.045) (Figure 5D). Raw data are available in Data Set 1 in Supplementary Material.

In the femur, 20-week HFD caused a significant decrease in cortical thickness relative to ND control (Figure 6D). Cortical tissue mineral density was also decreased with HFD (Figure 6F). With weight loss, cortical thickness and total mineral density improved relative to ND (Figures 6D,F). No differences in total area, marrow area, cortical area, or Iyy were noted (Figures 6A–F). Raw data are available in Data Set 2 in Supplementary Material.

### Marrow Adipose Tissue Expansion Correlates with Bone Loss in the Tibia

In the control ND group, pooled over all ages (12, 16, and 20 weeks of diet), there was a significant inverse correlation between MAT volume in the proximal metaphysis and measures of trabecular morphology including BVF, trabecular thickness, and BMC – but not with trabecular number (Figures 7A–D). By contrast, in the HFD group, MAT volume was negatively correlated with trabecular BVF, thickness, and BMC in addition to trabecular number (Figures 7A–D). Cortical thickness was significantly negatively correlated with GP to T/F J MAT volume in the HFD group only (Figure 7E). Cortical TMD did not correlate with MAT volume in either group (Figure 7F).

### High-Fat Diet Causes Persistent Decreases in Biomechanical Properties of the Femur

Four-point bending was performed to assess the biomechanical integrity of HFD and WL femurs. The femurs from the 20-week HFD and WL groups broke under a reduced maximum load relative to ND, trending toward less total work to induce fracture (Figures 8A,B). This indicates that despite recovery of cortical thickness and mineral density with WL (Figure 6), the bone quality remains impaired, leading to decreased fracture resistance and poor post-yield behavior. The yield load and post-yield work trended toward a decrease relative to ND in the HFD and WL groups, respectively (*p* < 0.1) (Figures 8D,E). The stiffness, post-yield displacement, and modulus of elasticity (39) were not significantly different between groups (Figures 8C,F) (Data Set 3 in Supplementary Material).

## Discussion

To our knowledge, this is the first study that has measured, within the same bone, HFD-induced MAT expansion and changes in skeletal morphology. By incorporating a weight loss group, we were also able to inhibit MAT expansion, and thus examine the impact of HFD on bone quality in the absence of MAT accumulation. In this study, HFD caused increases in body mass at 12-weeks, indicating accumulation of peripheral adiposity. This occurred prior to increases in MAT, supporting the hypothesis that dysfunction of peripheral tissues (e.g., insulin resistance) occurs prior to HFD-induced MAT expansion.

As adipocytes and osteoblasts arise from the same mesenchymal progenitor cell, the notion of “fate-switching” whereby one lineage is favored over the other has been suggested (29–31). The inhibition of osteoblast differentiation may subsequently lead to increased adipocyte production, thus presenting with a situation of reduced bone mass and increased MAT (44). It is of note that this concept fails to capture the complexity of skeletal progenitors – some of which have the capacity to differentiate into osteoblasts but not adipocytes (45). In our study we observed the well-documented inverse correlation between MAT volume and bone mass/density in the tibia (Figure 7). However, despite this correlation, our data do not support the hypothesis that MAT expansion is the sole mediator of bone loss with HFD. Specifically, deterioration of trabecular architecture occurred as early as 12-weeks after HFD in the tibia, while changes in MAT did not become statistically significant until 16 weeks (Figures 2 and 3). Furthermore, though switching from HFD to chow at 12-weeks completely prevented HFD-induced MAT accumulation in the WL group (Figure 3B), loss of trabecular number and corresponding increases in trabecular spacing beyond that of controls still occurred (Figures 3E,G). Thus, in this context, inhibition of MAT expansion by weight loss was not sufficient to block HFD-induced decreases in trabecular bone within the tibial metaphysis.

There are many MAT-independent effects with the potential to regulate bone during high-fat feeding, the presence of which may contribute to the cancellous and cortical bone loss observed in this model. Increased fat mass is associated with increased systemic markers of oxidative stress in both humans and mice (4). Increased peroxide (H_2_O_2_) and reduced endothelial nitric oxide synthase in a genetic model of obesity was associated with cancellous bone loss (46). Reactive oxygen species have been found to promote the association of the transcription factors FoxO with β-catenin, subsequently leading to a reduction in Wnt signaling and osteoblastic differentiation (47). Although the direct effects of leptin may also promote osteoblast proliferation and differentiation (25, 48), the central effects of leptin have been shown to mediate the opposite effects, promoting cancellous bone loss *via* the sympathetic nervous system (23, 24). Another central pathway involving increased neuropeptide Y (NPY) arising from leptin resistance during obesity is implicated in bone metabolism as mice with increased central NPY have concurrent obesity with bone loss (49) and NPY deficiency in *ob/ob* mice leads to improved cortical bone mass (50).

Lastly, there is an increase in systemic inflammation with obesity that might directly affect bone marrow osteoclasts. A major source of obesity-induced inflammation stems from an increase in bone marrow macrophages and their progenitors (51). These bone marrow-derived macrophages during obesity mediate an inflammatory environment that has been shown to stimulate osteoclastogenesis and reduce osteoblast development (52, 53), possibly due to the expansion of the common monocyte-osteoclast progenitor (54). Recently, Yue et al. have also demonstrated that leptin produced from obese adipose tissue can directly bind to leptin receptors on mesenchymal stem cells promoting differentiation of adipocytes and inhibiting osteoblast formation (29). Altogether there are a number of MAT-independent variables involved in coordinating the relationship between diet-induced obesity and bone.

Though it is not the sole mediator of bone loss with HFD, our study does not rule out the possibility that MAT, particularly when present in large excess, may exert detrimental effects on bone. Indeed, the magnitude of cancellous bone loss in the tibia (Figure 3) and cortical bone loss in the femur (Figure 6) was significantly greater in the 20-week HFD group with MAT expansion than the WL group in which MAT expansion failed to occur. Comparisons between the femur and tibia provide further clues as to this relationship. Consistent with a previous report, 12-weeks of HFD followed by 8-weeks of normal chow diet (WL group) did not prevent cancellous bone loss in the distal femoral metaphysis (12). By contrast, in the same animals, weight loss partially prevented HFD-induced deterioration of trabecular BVF and BMC in the tibia (Figure 3). It is possible that this discrepancy may be explained by differences in MAT. After 20-weeks of HFD, the volume of MAT in the metaphysis of the tibia was 9.8-fold greater than in the femur. This is similar to previous work by Halade et al., despite substantial differences in their model system (10% corn oil diet for 24-weeks in 12-month-old female mice) (44). Thus, it is possible that this increase in MAT contributed to additional bone loss in the tibia, beyond that observed in the femur, subsequently leading to a difference between the HFD and WL groups. However, given previous work, the nuances of this observation remain unclear (44).

Direct interactions between MAT and bone may influence bone loss during high fat feeding. Recently, MAT was found to be a significant contributor of circulating adiponectin during calorie restriction (55), this emphasizes the potential of MAT to influence not only bone but also whole body homeostasis. Direct adipose-bone pathways have been demonstrated to influence bone mass; the main two adipokines implicated are leptin (29, 48, 56–58) and adiponectin (59–61). More locally within the bone microenvironment, *in vitro* experiments have demonstrated that the release of free fatty acids from adipocytes inhibited osteoblast differentiation and promoted apoptosis through ROS production (62). Interestingly, co-cultures of osteoblasts and osteoclasts with adipocytes suggest that in addition to reducing osteoblastogenesis, osteoclastogenesis may be increased with increased adiposity, resulting in reduced bone mass (31).

Biomechanically, weight loss after 12-weeks of HFD was insufficient to rescue impaired fracture resistance. Indeed, the maximum load endured by the HFD and WL femurs was nearly identical – despite almost complete recovery of body mass and prevention of MAT expansion in the WL group. Comparable stiffness and modulus of elasticity in the ND, WL, and HFD groups indicates that the elastic properties of the bone were not affected. However, the failure properties were similarly reduced in both the HFD and WL groups, despite differential rescue of tissue mineral density and cortical thickness, implying that femur architecture fails to explain the impaired biomechanics. This may point to dysfunction within the organic properties of the bone, such as impaired cross-linking of collagen (26), as a potential mediator of persistent HFD-induced fracture risk.

Our study demonstrates that HFD causes long-term, persistent changes in bone quality. We started HFD at an age in which skeletal development is still highly active, likely contributing to impaired bone accrual during growth. Indeed, diet-induced obesity causes greater damage in growing bones (63). This is an important finding given the rise of obesity in pediatric populations (2, 64). Furthermore, these data demonstrate that MAT is not necessary for HFD-induced bone loss; however, MAT expansion, when present, may contribute to additional skeletal deterioration. It is likely that changes within the bone microenvironment including the adipocytes themselves are being altered but this was not examined in the current study (31, 65) and will need to be evaluated with future mechanistic investigations.

Given the rise in obesity across the age spectrum, this is a critical area of research and future studies are needed to determine the effects of weight loss (dietary or surgical) on bone density and to understand the mechanisms that drive changes in bone health. Even with the limitations a clear finding in this study is that there are some reversible and some permanent changes with HFD, followed by WL. Different regimens may be required to maintain bone health after WL, possibly with a focus on activity and diet (36).

## Author Contributions

ES and KS were involved in designing studies, completion of studies, data interpretation and analysis, and manuscript preparation. BK, KM, SK, KK, SA, and BZ were involved in completion of studies, data analysis, and reviewed the final manuscript.

## Conflict of Interest Statement

The authors declare that the research was conducted in the absence of any commercial or financial relationships that could be construed as a potential conflict of interest.
